# HIF-1α and Caspase-3 expression in aggressive papillary thyroid carcinoma

**DOI:** 10.1186/s12957-022-02815-8

**Published:** 2022-11-04

**Authors:** Lili Zhang, Baixue Shi, Mingyang Hu, Linxue Qian

**Affiliations:** Department of Ultrasound, Beijing Friendship Hospital, Capital Medical University, Beijing, China

**Keywords:** HIF-1α, Caspase-3, Thyroid carcinoma

## Abstract

**Background:**

Tumor cells adapt to hypoxia by regulating transcription factors that involved in regulation of metabolism, angiogenesis, cell proliferation, and apoptosis. Under hypoxic conditions, hypoxia-inducible factor-1 (HIF-1), consisting of HIF-1α and HIF-1β subunits, acts as a key transcription factor mediating the adaptive cellular responses. Caspase-3 is a key apoptosis-related protease that plays a role in tumor growth and development. Studies have shown that caspase-3 could be regulated by HIF-1α under pathological conditions. Therefore, HIF-1α and caspase-3 expression may be related to the poor prognosis of tumors. In this study, we analyzed the possible relationships between these two signaling factors in correlation with the clinical behavior of PTC.

**Methods:**

We detected the expression levels of HIF-1α and caspase-3 in 70 samples of PTC and para-cancerous tissues (control group) by immunohistochemistry (IHC). Furthermore, various clinicopathological parameters were assessed to determine their correlations with HIF-1α and caspase-3 expressions.

**Results:**

First, HIF-1α and caspase-3 expressions (60% and 37.1%, respectively) increased significantly in the PTC samples as compared to normal tissues (2.9% for both HIF-1α and caspase-3) (*p* < 0.05) as determined by IHC. Second, although there was no significant difference between the expression of HIF-1α and caspase-3 in regard to gender, age distribution, tumor size, lymph node metastasis, and *BRAF*^V600E^ mutation (all *p* > 0.05), HIF-1α and caspase-3 expressions were associated with capsule invasion and cell subtypes of PTC (*p* < 0.05). The percent positivity of caspase-3 expression in tall-cell variant (TCV) was the highest (63.6%). Third, HIF-1α expression was positively correlated with that of caspase-3 (*r*_s_ = 0.326; *p* < 0.05).

**Conclusions:**

Overexpression of HIF-1α and caspase-3 is associated with carcinogenesis. These factors might serve as promising predictors of aggressive PTC. These findings also suggest their potential as therapeutic targets.

## Background

Thyroid cancer is one of the most commonly seen endocrine malignancies, with an increasing annual incidence rate worldwide [1]. The majority of thyroid cancers originate from follicular epithelial cells. Of these subtypes, papillary thyroid carcinoma (PTC) accounts for about 85% of the overall cases. Although PTC carries a 5-year survival rate of 97%, about 15% of patients who relapse present with clinical symptoms of aggressive PTC, such as local tissue infiltration, cervical lymph node metastasis (CLNM), and even distant metastasis at an early stage [2–4]. Therefore, it is crucial to diagnose and treat aggressive PTC at the early stage of symptomatic manifestations.

The tumor microenvironment (TME) plays a significant role in the growth and metastasis of most cancers. Hypoxic condition within the TME has been found to be crucial in different stages of tumor development. Under hypoxic conditions, tumor cells adapt to hypoxia by transcription factors that involved in regulation of metabolism, angiogenesis, cell proliferation, and apoptosis. Hypoxia-inducible factor-1 (HIF-1), consisting of HIF-1α and HIF-1β subunits, is a key transcription factor that mediates these adaptive cellular responses [5]. Liu et al. have found that the decrease in cellular oxygen level is related to the poor prognosis of PTC patients [6]. Therefore, the induction of HIF-1α expression can promote the aggressiveness of tumors.

However, the underlying molecular mechanisms are not fully understood. Some studies have reported that the transcription of several apoptosis-related genes, including survivin, Bcl-2, and caspase-3, could be regulated by HIF-1α [7, 8]. Caspase-3 is shown to act as a key protease during apoptosis as well as in tumor growth and development. To date, none of the studies has systematically assessed the expression profiles of both HIF-1α and caspase-3 and their associated signaling pathways in PTC.

Therefore, we aimed to analyze the expressions of HIF-1α and caspase-3 to reveal the possible cross talk between these two factors and explore their correlations with the patho-clinical behaviors of PTC.

## Methods

### Patients and specimens

This study recruited 80 PTC patients who received thyroidectomy at our hospital, between January 2019 and June 2019. We excluded 10 patients with Hashimoto thyroiditis. Biopsies of the remaining 70 PTC patients were subjected to immunohistochemistry (IHC) to assess HIF-1α and caspase-3 expressions, and the relatively normal para-cancerous tissues were used as controls. The carcinoma samples were additionally analyzed to detect the *BRAF*^V600E^ mutation.

The inclusion criteria were as follows: (1) patients did not receive any treatments prior to thyroidectomy; (2) ultrasound data was complete; (3) PTC diagnosis, capsule invasion, and CLNM were pathologically confirmed; (4) the absence of any other carcinomas was confirmed; (5) sufficient amount of DNA were extracted from biopsy samples to detect the *BRAF*^V600E^ mutation; and (6) patients who had total thyroidectomy underwent the central neck dissection.

This study was approved by the ethics committee of our hospital. As this is a retrospective study, informed consent was waived.

### Cell subtype determination

All formalin-fixed paraffin-embedded (FFPE) tumor tissues were sliced into 4 μm sections, and one slide for each tissue sample was subjected to the hematoxylin and eosin staining. All histological slides were reviewed by pathologists to identify cell subtypes of PTC, including tall-cell variant (TCV), classic variant of papillary thyroid carcinoma (CVPTC), and follicular variant of papillary thyroid carcinoma (FVPTC), by a light microscope. The tumor area was marked to extract genomic DNA.

### Analysis of the BRAF^V600E^ mutation

Genomic DNA was extracted from paraffin-embedded tissues of PTC patients. Under the manufacturer’s instructions, the process was performed using an FFPE DNA Kit (Cat No. ADx-FF01, Xiamen, China). The A260:A280 values ranged from 1.7 to 1.9. The DNA samples were then subjected to the next-generation sequencing (NGS) to determine BRAF^V600E^ genotypes (Beijing Jingkerui Biotechnology Co., Ltd., Beijing, China).

### Immunohistochemistry staining

Paraffin-embedded blocks were sectioned to 4 μm thickness. After de-paraffinization and rehydration, antigen retrieval was carried out by boiling the tissue slide in EGTA antigen retrieval solution (Zhongshan Golden Bridge Biotechnology, China, pH 8.0) and then washing the section with phosphate-buffered saline (PBS). Thyroid sections were incubated with rabbit antihuman HIF-1α (1:200, Zhongshan Golden Bridge Biotechnology, China) and rabbit anti-human caspase-3 (1:500, Cell Signaling Technology, Danvers, MA, USA) at 4 °C overnight. After three washes with PBS, secondary antibodies (Maixin Biotechnology Co., Ltd.) were added to the sections, followed by counterstaining with hematoxylin and observation with a microscope. All slides were reviewed and evaluated by three pathologists who were blinded to the clinical data.

Stromal staining of HIF-1α and caspase-3 expressions was graded using a quick score (QS) system. The QS represents the sum of a proportional score (PS) and intensity score (IS). The PS was classified into five levels: 0: 0–5%; 1: 6–25%; 2: 26 to 50%; 3: 51–75%; 4: 76 to 100%, while the IS was graded as follows: −(0), +(1), ++(2), and +++(3). The QS was defined as follows: 0–3 scores were regarded as negative and 4–7 scores as positive [9].

### Statistical analysis

Statistical analyses were performed using the SPSS version 16.0 (SPSS, Inc., Chicago, USA). Pearson’s chi-square test was used for categorical variables. The correlation between HIF-1α and caspase-3 expressions was evaluated by Spearman correlation analysis. A *p*-value of < 0.05 was considered statistically significant. To identify risk factors for capsule invasion in PTC, a multivariate logistic regression analysis was performed.

## Results

### Expressions of HIF-1α and caspase-3 in PTC and normal tissues

**Fig. 1:**
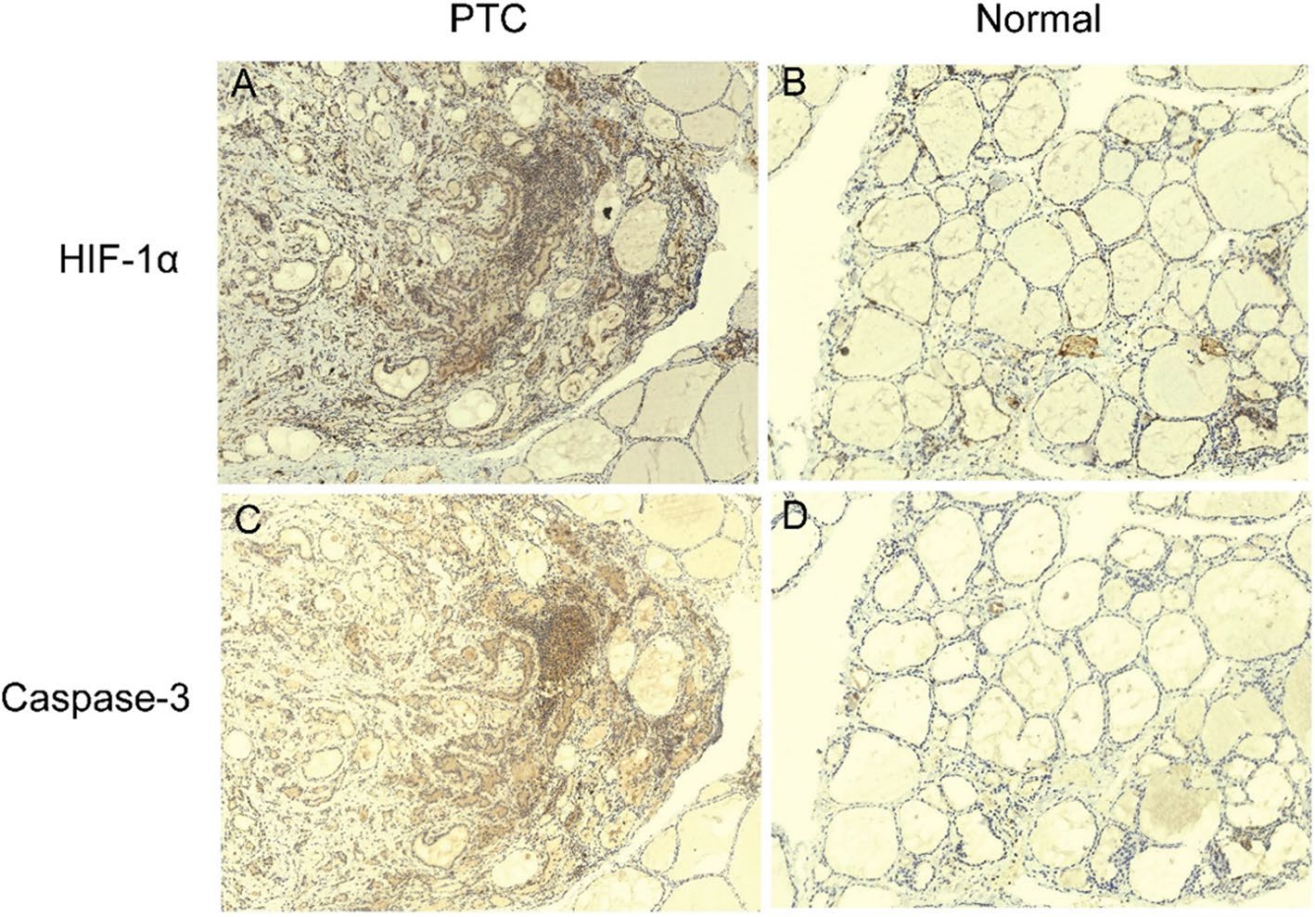
Representative images of HIF-1α and caspase-3 expression in papillary thyroid carcinoma (PTC). **A** Expression of HIF-1α in PTC. **B** Expression of HIF-1α in normal tissues. **C** Expression of caspase-3 in PTC. **D** Expression of caspase-3 in normal tissues. Magnification is ×100

Both HIF-1α and caspase-3 expressions in 70 PTC samples and their relatively normal para-carcinoma tissues were detected by IHC. The positive staining of HIF-1α and caspase-3 was located in the cytoplasm (Fig. 1). The results showed that HIF-1α and caspase-3 expressions (60% and 37.1%, respectively) increased significantly in the PTC samples as compared to that in normal tissues (both expression levels were 2.9%) (*p* < 0.05; Fig. 1 and Table 1). This indicated that HIF-1α and caspase-3 were involved in the carcinogenesis of PTC.

**Table 1:** HIF-1α and caspase-3 expression in PTC and normal tissues

	PTC, *n* (%)	Normal, *n* (%)	*p*-value
HIF-1α			0.000*
Positive	42 (60)	2 (2.9)	
Negative	28 (40)	68 (97.1)	
Caspase-3			0.000*
Positive	26 (37.1)	2 (2.9)	
Negative	44 (62.9)	68 (97.1)	

*p < 0.05

### Correlations of HIF-1α and Caspase-3 expressions with clinicopathological parameters

A total of 70 consecutive patients, including 58 females and 12 males, were used for data analysis. The mean age was 44 ± 12 years. There were no significant differences between HIF-1α and caspase-3 expressions with respect to gender, age distribution, and tumor size (all *p* > 0.05; Table 2). Additionally, HIF-1α expression was significantly associated with capsule invasion (*p* < 0.05; Table 2). However, caspase-3 expression indicated no notable correlation with capsule invasion (*p* > 0.05, Table 2).

**Table 2:** Clinicopathological features of patients with PTC

Variable	HIF-1α expression, *n* (%)		*p*-value	caspase-3 expression, *n* (%)		*p*-value
	Positive	Negative		Positive	Negative	
Number	42 (60)	28 (40)		26 (37.1)	44 (62.9)	
Gender			0.650			0.978
Female	36 (62.1)	22 (37.9)		21 (36.2)	37 (63.8)	
Male	6 (50.0)	6 (50.0)		5 (41.7)	7 (58.3)	
Age (year)			0.557			0.660
< 45	24 (63.2)	14 (36.8)		15 (39.5)	23 (60.5)	
≥ 45	18 (56.2)	14 (43.8)		11 (34.4)	21 (65.6)	
Tumor size (cm)			0.626			0.322
< 1	22 (62.9)	13 (37.1)		11 (31.4)	24 (68.6)	
≥ 1	20 (57.1)	15 (42.9)		15 (42.9)	20 (57.1)	
Capsule invasion			0.027*			0.247
Yes	33 (68.8)	15 (31.2)		20 (41.7)	28 (58.3)	
No	9 (40.9)	13 (59.1)		6 (27.3)	16 (72.7)	
CLNM			0.768			0.083
Yes	18 (58.1)	13 (41.9)		15 (48.4)	16 (51.6)	
No	24 (61.5)	15 (38.5)		11 (28.2)	28 (71.8)	
*BRAF*^V600E^mutation	17 (73.9)	6 (26.1)	0.096	9 (39.1)	14 (60.9)	0.810
PTC subtype			0.238			0.014*
FVPTC	20 (58.8)	14 (41.2)		7 (20.6)	27 (79.4)	
CVPTC	13 (52.0)	12 (48.0)		12 (48.0)	13 (52.0)	
TCV	9 (81.8)	2 (18.2)		7 (63.6)	4 (36.4)	

*CLNM* cervical lymph node metastasis, *PTC* papillary thyroid carcinoma, *CVPTC* classic variant of papillary thyroid carcinoma, *FVPTC* follicular variant of papillary thyroid carcinoma, *TCV*, tall-cell variant

*p < 0.05

Both LNM and *BRAF*^V600E^ mutation exhibited no significant differences in HIF-1α expression levels (all *p* > 0.05; Table 2), as well as in caspase-3 levels (all *p* > 0.05; Table 2).

Furthermore, the level of caspase-3 expression was specific to PTC cell type (*p* < 0.05; Table 2). The percent positivity of caspase-3 expressions in TCV, CVPTC, and FVPTC was 63.6%, 48%, and 20.6%, respectively.

However, HIF-1α expression showed no significant difference among different PTC cell subtypes (*p* > 0.05; Table 2).

### The correlative relationship between HIF-1α and caspase-3 expression

The correlation between HIF-1α and caspase-3 expression was evaluated by the Spearman correlation analysis as listed in Table 3. The positive expression of HIF-1α was significantly associated with that of caspase-3 (*r*_s_ = 0.326; *p* < 0.05; Table 3).

**Table 3:** Association between HIF-1α and caspase-3 expression

HIF-1α	Caspase-3		*r*_s_	*p*-value
	Positive	Negative		
Positive	21	21	0.326	0.006*
Negative	5	23		

*p < 0.05

### Univariate analysis for capsule invasion in PTC

The univariate analysis of clinicopathological features revealed that tumor size and HIF-1α-positive expression were significantly different between PTCs with and without capsule invasion (*p* < 0.05; Table 4). However, no significant difference was detected between the two groups in gender, age, *BRAF*^V600E^ mutation, cell subtypes, and caspase-3 expression (all *p* > 0.05, Table 4).

**Table 4:** Predictive factors of capsule invasion in PTC

Parameter	PTCs with/without capsule invasion		*p*-value
	Yes (*n* = 48)	No (*n* = 22)	
Gender			0.128
Female	42 (72.4%)	16 (27.6%)	
Male	6 (50.0%)	6 (50.0%)	
Age (year)			0.288
< 45	24 (63.2%)	14 (36.8%)	
≥ 45	24 (75.0%)	8 (25.0%)	
Tumor size (cm)			0.002*
< 1	18 (51.4%)	17 (48.6%)	
≥ 1	30 (85.7%)	5 (14.3%)	
*BRAF*^V600E^ mutation	16 (69.6%)	7 (30.4%)	0.900
PTC subtype			0.686
FVPTC	25 (73.5%)	9 (26.5%)	
CVPTC	16 (64.0%)	9 (36.0%)	
TCV	7 (63.6%)	4 (36.4%)	
HIF-1α^+^	33 (78.6%)	9 (21.4%)	0.027*
Caspase-3^+^	20 (76.9%)	6 (23.1%)	0.247

*p < 0.05

## Discussion

In most cases, PTC is regarded as an indolent tumor which has a favorable prognosis. However, cell subtype, tumor size, and the expression of carcinoma markers may affect the prognosis, while the underlying molecular pathomechanism remains elusive so far. This study identified that cytoplasmic expression of HIF-1α was present in 60% of PTC patients, compared to para-carcinoma tissues (2.9%), which was consistent with previous reports [10, 11]. This indicated that HIF-1α might play important transcriptional and regulatory roles at an early stage of PTC.

Recent studies have revealed that HIF-1α expression is upregulated in solid tumors such as colorectal carcinoma [12], hepatocellular carcinoma [13], and thyroid cancer [14]. Although PTC has a mortality rate of less than 5%, there is still a small number of patients with metastasis or relapse. HIF-1α, as one of the HIF family members, is critically related to tumor metastasis and tumor staging [15]. In PTC, increased HIF-1α level is associated with CLNM and distant metastasis [6, 16]. Our study assessed relationships between the HIF-1α expression and several clinicopathological parameters in PTC patients. As a result, HIF-1α expression showed no significant differences among PTC patients in terms of gender, age distribution, and tumor size, which were consistent with previous findings [6]. Pathological type and *BRAF*^V600E^ mutation status were considered important prognostic factors for PTC patients. We analyzed the correlations between cell subtypes, *BRAF*^V600E^ mutation, and HIF-1α expression and found that there was no significant association of the proportion of cell subtypes with *BRAF*^V600E^ mutation status.

Like other tumors, thyroid carcinoma has biological characteristics of infiltration and metastasis, even breaking through the capsule into surrounding nerves and muscles. This study showed high levels of HIF-1α expression in PTC patients with capsule invasion. Previous studies have confirmed that capsule invasion is one of the major risk factors for LNM [17, 18]. Thus, this study suggests that HIF-1α expression may be related to LNM pathogenesis. Unfortunately, LNM showed no significant differences with or without HIF-1α expression, which was contradictory to a previous report [6], and that might be due to the small sample size used in our study. Together, these results suggest that HIF-1α induction may be involved in the invasion of PTC. Whether HIF-1α expression correlates with metastasis in PTC needs further investigations. Some clinical features, such as TNM staging and distant metastasis, should be included in the prognosis of PTC patients.

Recent studies indicate that HIF-1α regulates invasion, metastasis, and prognostic evaluation in PTC through various mechanisms [11, 16, 19]. As we know, solid tumors generally possess hypoxic microenvironments during their development. Under hypoxic conditions, HIF-1α could bind to HIF-1β to form the HIF-1 complex that can promote the transcription of downstream genes involved in angiogenesis, tumor invasion, and metastasis by binding to the hypoxia response element (HRE) of the gene promoters to regulate their expressions [20]. Thus, HIF-1α upregulates the expression of these genes to promote invasion and metastasis of PTC.

Caspase-3 has been reported as a key protease in the apoptosis process [21]. Recently, Huang et al. have found that the high level of caspase-3 in patients is associated with head and neck cancers [22]. Thyroid cancer is one of the common head and neck cancers. There are a few research studies on the caspase-3 expression in thyroid cancer. In this study, the expression of caspase-3 was significantly associated with the occurrence of PTC. Notably, the caspase-3 expression did not show any significant differences in gender, age distribution, tumor sizes, and the *BRAF*^V600E^ mutation status. However, caspase-3 expression was significantly associated with the PTC subtype. It was higher in TCV (63.6%) than in any other cell subtype. About 20.6% of FVPTC patients presented caspase-3-positive expression. This indicated that caspase-3 might be related to aggressive tumor behavior and prognosis and was consistent with previous studies [23, 24]. LNM is considered a significant aggressive tumor behavior. Therefore, we analyzed the caspase-3 expression in PTC patients with LNM. Possibly due to the small sample size, we could not detect any significant difference in caspase-3 expression between LNM and non-LNM groups. Furthermore, HIF-1α expression was positively associated with that of caspase-3 in PTC (*r*_s_ = 0.326; *p* < 0.05). However, there were no significant differences in caspase-3 expressions in PTC patients with capsule invasion.

These results suggest that caspase-3 expression may correlate to aggressive behavior in PTC. PTC patients with high caspase-3 expressions may be at higher risks for LNM, distant metastasis, and low survival rates. These parameters should be included to analyze the relationship between caspase-3 and clinical prognosis in future studies. Recently, it has been shown that caspase-3 could promote oncogenic transformation by inducing genetic instability [25]. This might explain why caspase-3 expression was high in TCV. The molecular mechanism remains to be further explored.

Therefore, HIF-1α and caspase-3 might be used as potential biomarkers for the diagnosis as well as therapeutic targets for aggressive PTC cases. Moreover, we found that tumor sizes of ≥ 1.0 cm along with increased HIF-1α expression were independent predictors of the risk of capsule invasion. Further studies are warranted to explore predictors of capsule invasion.

There are several limitations in this study. First, it was a single-institution retrospective observational study, which might have selection bias. Second, the sample size was not statistically large; therefore, we could not arrive at a definitive conclusion about the involvement of LNM and *BRAF*^V600E^ mutation in PTC aggression and prognosis. A large-scale study will be necessary to determine the exact pathological roles of these molecules in the diagnosis and prognosis of PTC. Third, survival and death of PTC patients were not analyzed in our study; therefore, we could not observe their hard points. We will collect more cases and track their hard points to evaluate their prognostic value in our post-study.

## Conclusions

Overexpression of HIF-1α and caspase-3 could be associated with PTC. For the first time, we reported the positive correlation between HIF-1α and caspase-3 expression in PTC. They are promising predictors of aggressive PTC and may serve as new therapeutic targets for PTC treatment.

## Data Availability

All data generated or analyzed during this study are included in this published article.

## Abbreviations

HIF-1α: Hypoxia-induced factor 1α
PTC: Papillary thyroid carcinoma
IHC: Immunohistochemistry
TME: Tumor microenvironment
CLNM: Cervical lymph node metastasis
FFPE: Formalin-fixed paraffin-embedded
CVPTC: Classic variant of papillary thyroid carcinoma
FVPTC: Follicular variant of papillary thyroid carcinoma
TCV: Tall-cell variant
NGS: Next-generation sequencing
PBS: Phosphate-buffered saline
QS: Quick score
PS: Proportional score
HRE: Hypoxia response element
